# From Muscular Hypertonus to Equilibrium: A Conceptual Framework for Aesthetic Neuromodulation Based on the Index of Muscular Equilibrium (IME)

**DOI:** 10.3390/toxins18020115

**Published:** 2026-02-23

**Authors:** Andrea Felice Armenti

**Affiliations:** LA VISION Training Institute, 00185 Rome, Italy; andrea.armenti@lavisiontin.com

**Keywords:** botulinum toxin type A, facial muscles, facial expression, hypertonia, aesthetics, neuromodulation, treatment outcome, clinical decision-making, photogrammetry, conceptual framework

## Abstract

Facial neuromodulation with botulinum toxin has traditionally been approached from the perspective of wrinkle correction. However, facial expressions primarily arise from coordinated muscular interactions that convey both positive and negative emotional valence. A conceptual framework focused on muscular equilibrium rather than wrinkle severity may therefore offer a more comprehensive, reproducible, and clinically meaningful approach. In this article, we propose the Index of Muscular Equilibrium (IME) Framework, a conceptual model for aesthetic neuromodulation that integrates functional muscle mapping, validated severity scales, and a composite IME score to support personalized treatment planning and outcome assessment. The framework is derived from a narrative review of PubMed-indexed literature on facial muscle activity, emotional expression, and validated clinical assessment tools. It combines a Valence Map to classify positive- and negative-valence muscle groups, a standardized evaluation of static and dynamic hypertonus, a conceptual Plan Score to guide selective neuromodulation, and a feedback-based longitudinal workflow (the IME Loop). Together, these components enable structured assessment of muscular imbalance, integration of established wrinkle severity scales, and translation into individualized, function-oriented treatment strategies, with intended benefits including improved objectivity, reproducibility, and patient communication. By reframing treatment success from the duration of muscle blockade to the duration of expressive harmony, the IME Framework introduces testable constructs for future validation and offers a functional perspective on facial neuromodulation aligned with contemporary affective science.

## 1. Introduction

Facial expression is a primary channel of nonverbal communication that emerges from the coordinated activity of multiple facial muscles rather than from cutaneous lines alone. In this view, emotional valence—the positive or negative quality of affect—is reflected in distinct patterns of muscle recruitment: increased activity of depressor muscles such as the corrugator supercilii is consistently associated with negative affective states, while activation of elevators including the zygomaticus major supports positive affiliative signaling [1,2]. Facial muscle movements were referenced using the Facial Action Coding System (FACS), a standardized taxonomy of Action Units originally developed by Ekman and colleagues and extensively reviewed in contemporary methodological work [3,4]. Based on this evidence, muscular equilibrium can be defined as a functional balance between muscle groups that transmit negative versus positive-valence signals, producing facial output that appears natural and congruent with the intended social message of the individual.

Aesthetic neuromodulation with botulinum toxin offers the opportunity to modulate these muscular interactions. However, clinical decision-making would benefit from a structured, measurable model that (i) captures hypertonus at rest and during expression, (ii) acknowledges the communicative role of positive-valence elevators, and (iii) translates observation into reproducible indices suitable for longitudinal evaluation and patient communication.

Here we present the IME Framework (Index of Muscular Equilibrium Framework), designed to operationalize these principles. The framework consists of four integrated components: a Valence Map that classifies facial muscles by their contribution to emotional valence; an Index of Muscular Equilibrium (IME) that synthesizes static/dynamic hypertonus and validated wrinkle-severity scales into a standardized score; a Personalized Plan Score that links IME domains to selective neuromodulation strategies; and the IME Loop, a feedback-based workflow for longitudinal monitoring and adjustment.

In this context, hypertonus refers to visible muscular overactivity, either at rest or during expression, manifesting itself as contraction or distortion beyond the intended communicative range. Together, these components aim to provide a reproducible, clinically significant pathway from functional assessment to personalized intervention. Although developed in the context of aesthetic neuromodulation, this framework is intentionally aligned with broader domains of facial medicine, where botulinum toxin is used to treat functional facial conditions characterized by muscular imbalance, hyperactivity, or altered expression.

The objective of this article is to describe the theoretical rationale, structure, and measurement approach of this framework, establishing clear, testable constructs for future psychometric and clinical validation.

## 2. Methods

### 2.1. Study Design and Scope

This work presents a conceptual framework for aesthetic neuromodulation with botulinum toxin, designed to emphasize muscular equilibrium and emotional valence in both aesthetic and functional facial contexts. Patient data were not collected, and all examples presented are hypothetical. The framework is intended to provide clearly defined, testable constructs for subsequent validation in prospective clinical studies.

The framework was developed through a narrative review of PubMed-indexed literature, focusing on facial anatomy, affective neuroscience, validated aesthetic assessment scales, and international consensus guidelines on botulinum toxin use [5,6,7]. Based on this synthesis, the resulting model is structured as a reproducible system composed of four integrated components:

1. The Valence Map;
2. Standardized assessment of muscular hypertonus and wrinkle severity;
3. The Index of Muscular Equilibrium (IME);
4. The IME Loop for longitudinal monitoring and adaptive treatment refinement.

### 2.2. Standardized Acquisition Protocol

Facial dynamics is evaluated using standardized photographic and video acquisition under controlled conditions, including fixed lighting, neutral head position aligned to the Frankfort horizontal plane, and constant camera distance [5]. This standardized setup is intended to minimize technical variability and ensure reproducibility across assessments.

Static facial documentation includes the following views:

- Frontal;
- Three-quarters right;
- Three-quarters left.

Dynamic facial assessment is performed using a predefined sequence of tasks designed to elicit specific muscle group activation patterns:

- Neutral repose maintained for 3–4 s;
- Frown on command (“corrugate your brows”);
- Closed-lip smile to assess zygomaticus activation and periocular coupling;
- A broad smile with teeth to evaluate the balance between the depressor anguli oris and the zygomaticus muscles;
- Eyebrow elevation (“raise your eyebrows”);
- Forced grimacing or mandibular depression to induce platysmal activation.

Dynamic activation of the platysma is specifically assessed during forced grimacing or mandibular depression tasks, as these maneuvers reliably induce the prominence of the platysmal band and caudal depressor activity, which have been shown to influence lower facial contour and aesthetics of the jawline [8].

To reduce inter- and intra-subject variability, participants receive standardized verbal instructions and perform brief practice trials before image and video acquisition. Quantitative measurements are recorded in millimeters (mm) or degrees (°) using digital calipers, calibrated grids, or augmented-reality measurement tools.

### 2.3. Assessment of Hypertonus

Validated photonumeric scales widely used in aesthetic medicine primarily quantify cutaneous manifestations, such as wrinkle depth and distribution [6,7]. However, these instruments do not capture the underlying muscular hyperactivity, which represents the direct biological target of neuromodulation with botulinum toxin. As a result, although the skin results are systematically assessed, the clinical evaluation of facial muscular hypertonus—both at rest and during voluntary expression—is not sufficiently standardized in current aesthetic practice for most facial mimetic muscles. An exception is platysma, for which validated photonumeric classification scales of the severity of the platysmal band have been developed and have demonstrated good reliability between and between rats [9].

To address this methodological gap, three complementary assessment instruments are introduced as conceptual tools: the Face Repose Tension Scale (FRS), the Facial Dynamics Hypertonus Scale (FDHS) and the Eyebrow Symmetry and Position Score (ESPS). These scales are intended to provide structured and observable descriptors of muscular overactivity and asymmetry under static and dynamic conditions. At this stage, they are explicitly proposed as components of the IME Framework and are presented as candidate measures for future psychometric validation.

### 2.4. Static and Dynamic Hypertonus Assessment

#### 2.4.1. Static Hypertonus (Repose)

Static hypertonus is defined as visible muscle contraction at rest, including persistent glabellar scarring, downward displacement of the oral commissures, or baseline facial asymmetry. This condition reflects tonic muscular overactivity in the absence of voluntary expression. In the cervical–mandibular region, static hypertonus can also manifest itself as visible platysmal banding at rest, which can be assessed using validated photonumeric platysmal band severity scales.

Static hypertonus is assessed using the Facial Repose Tension Scale (FRS), a five-point ordinal scale ranging from 0 (no visible tension) to 4 (marked and persistent tension).

The FRS is newly proposed within the IME Framework and is intended as a conceptual assessment tool. Psychometric validation, including reliability and construct validity testing, will be required in future clinical studies.

#### 2.4.2. Dynamic Hypertonus (Expression Tasks)

Dynamic hypertonus is defined as excessive muscle contraction or distortion that occurs during standardized facial movements. It reflects hyperactive recruitment beyond the intended communicative range during voluntary expression. Dynamic platysmal activation during forced grimacing or mandibular depression represents an additional source of negative-valence expression and can be graded using established platysmal band scales [9].

Dynamic hypertonus is assessed using the Facial Dynamics Hypertonus Scale (FDHS), a five-point ordinal scale (0–4), applied during predefined expression tasks. Scoring incorporates both:

- contraction amplitude, quantified in millimeters (mm) or degrees (°);
- severity of distortion, such as exaggerated depth of the glabellar groove or excessive commissural descent.

The FDHS is introduced here as a conceptual instrument within the IME Framework and is explicitly proposed for future psychometric validation.

#### 2.4.3. Wrinkle Severity Assessment

Validated photonumeric scales were integrated to complement hypertonus scoring by recording established cutaneous manifestations of muscular activity [5,6,7]. The following region-specific instruments were used:

- glabella: Merz Glabellar Line Severity Scale (GLSS; 0–4);
- periocular region (“crow’s feet”): Crow’s Feet Severity Scale (CFSS; 0–4);
- forehead: Forehead Line Severity Scale (FLSS; 0–4);
- commissure/depressor anguli oris region: commissural position assessed using angular measurements (°), informed by geometric facial analysis methods [10].

Each facial region is evaluated at rest and during maximal voluntary contraction.

### 2.5. Eyebrow Position and Symmetry

The Eyebrow Symmetry and Position Score (ESPS) is introduced to assess upper-face equilibrium, reflecting the balance between elevators (frontalis) and depressors (corrugator, orbicularis oculi, procerus). ESPS is assessed across three anatomical segments:

- medial head;
- central body;
- lateral tail.

For each segment, the position is quantified as the vertical distance from the orbital rim to the brow margin (mm). Symmetry is operationalized as the left–right difference for each segment. ESPS is scored from 0 (marked asymmetry and/or ptosis) to 3 (optimal elevation and symmetry), with the interpretation explicitly contextualized to population-specific anatomical norms. The baseline eyebrow position is known to vary between ethnic groups, age, and sex, and therefore the ESPS values should be interpreted in relation to appropriate demographic reference standards rather than fixed anthropometric ideals.

ESPS is a metric proposed within this conceptual model and requires future psychometric validation.

The FRS, FDHS, and ESPS scales are newly proposed within this framework. They are introduced for conceptual purposes and require future psychometric validation, including inter- and intra-rater reliability, test–retest stability, and convergent validity against established patient-reported outcome measures (e.g., FACE-Q Aesthetics) [11]. All assessment instruments integrated into the IME Framework are summarized in Table 1.

### 2.6. Index of Muscular Equilibrium (IME)

#### 2.6.1. Negative-Valence Domains

Negative-valence domains are restricted to facial mimetic regions directly involved in emotional signaling; therefore, cervico-facial muscles such as the platysma are not included as computational domains within the IME formulation. This restriction reflects a conceptual distinction rather than an anatomical exclusion. Muscles such as the platysma or mentalis may significantly influence facial appearance and biomechanical context; however, within the IME framework, they are treated as extracore modulators that inform clinical interpretation and treatment planning without directly encoding discrete emotional valence. Their inclusion in the IME computation would risk conflating contextual contour modulation with expressive equilibrium.

Three domains—the glabella, periocular, and oral commissure—are modeled as negative-valence regions, in which increased activity is hypothesized to convey tension, fatigue, or sadness. For each domain *d*, the following quantities are defined:

(1) $${\mathrm{Hypertonus}}_{d}={\mathrm{FRS}}_{d}+{\mathrm{FDHS}}_{d}$$

where the resulting values range from 0 to 8.

(2) $${\mathrm{LineSeverity}}_{d}={\mathrm{ValidatedScale}}_{d}$$

where the resulting values range from 0 to 4.

(3) $${\mathrm{IME}}_{d}=0.6\cdot \frac{{\mathrm{Hypertonus}}_{d}}{8}+0.4\cdot \frac{{\mathrm{LineSeverity}}_{d}}{4}$$

For clarity, domain-level subscores (IMEd) are expressed as normalized values (0–1).

#### 2.6.2. Positive-Valence Domain: Frontalis–Eyebrow Complex

The frontal–brow complex is explicitly represented to acknowledge its putative role in signaling openness and attentiveness. Unlike lower-face or cervico-facial muscles, the frontalis–eyebrow complex is directly involved in rapid affective signaling and perceptual judgments of openness and engagement. Its inclusion is supported by evidence linking eyebrow elevation and upper-face dynamics to affective communication [2,12].

This domain combines three components:

1. Frontalis Mobility Score (FMS): mean normalized elevation in head, body, and tail segments (capped at 12 mm)
2. Forehead Display Score (FD_Skin_): an inverse transformation of forehead wrinkle severity
  (4) $${\mathrm{FD}}_{\mathrm{Skin}}=1-\frac{\mathrm{FLSS}}{4}$$
3. ESPS normalization:
  (5) $${\mathrm{ESPS}}_{\mathrm{norm}}=\frac{\mathrm{ESPS}}{3}$$
  Unlike hypertonus and wrinkle severity scales, ESPS is oriented such that higher values reflect improved positional equilibrium; its normalized contribution therefore increases the positive-valence domain score.

The resulting positive-valence domain score is computed as:

(6) $${\mathrm{IME}}_{\mathrm{Frontalis}}=0.50\cdot \mathrm{FMS}+0.25\cdot {\mathrm{FD}}_{\mathrm{Skin}}+0.25\cdot {\mathrm{ESPS}}_{\mathrm{norm}}$$

#### 2.6.3. Global IME

The four domains are integrated into a composite IME, scaled to 0–100:

(7) $$\begin{array}{cc} \mathrm{IME}=100\;\times & (0.30\cdot {\mathrm{IME}}_{\mathrm{Glabella}}\\ & +0.20\cdot {\mathrm{IME}}_{\mathrm{Periocular}}\\ & +0.40\cdot {\mathrm{IME}}_{\mathrm{Commissure}}\\ & +0.10\cdot {\mathrm{IME}}_{\mathrm{Frontalis}}) \end{array}$$

The composite IME is reported on a 0–100 scale for clinical interpretation and visualization.

The proposed domain weighting scheme is reported in Table 2 and should be interpreted as a set of theoretically informed priors, hypothesis-generating, rather than validated or definitive parameters. The relative weights assigned to each domain should be interpreted as theoretically informed initial priors, derived from psychophysiological evidence and clinical importance, rather than as fixed or definitive parameters. These weights are intentionally presented in explicit mathematical form to ensure transparency and to facilitate empirical refinement in future validation studies.

Consequently, IME should be interpreted as an index of expressive equilibrium rather than a comprehensive measure of facial rejuvenation or aesthetic improvement. Importantly, the added value of the IME does not lie in replacing expert qualitative assessment, but in integrating heterogeneous observations into a standardized structure. By combining static and dynamic hypertonus with cutaneous severity through explicit weighting, the IME allows comparable interpretation across domains, patients, and time points, while preserving domain-level subscores for differential prioritization.

### 2.7. Population Calibration Factor

Although the IME is presented as a universal index, facial expression and perceived emotional valence are known to vary according to cultural background, age, and gender at the population level. This variability includes differences in the position of the eyebrows at baseline and the morphology of the upper-face between ethnic groups, which may influence the perception of emotional valence. The population calibration factor is intended to account for such systematic differences in future validation studies. To account for this variability, the framework allows the application of a population calibration coefficient ($C_{f}$), derived from normative demographic datasets. When population calibration is applied, ${\mathrm{IME}}_{\mathrm{raw}}$ indicates the unadjusted global score and ${\mathrm{IME}}_{\mathrm{final}}$ the calibrated value.

The calibrated IME is defined as:

(8) $${\mathrm{IME}}_{\mathrm{final}}={\mathrm{IME}}_{\mathrm{raw}}\times C_{f}$$

where $C_{f}$ represents a correction factor (e.g., in the range of 0.9–1.1 for illustrative purposes) that adjusts the global IME score according to the validated population norms. At the present conceptual stage, $C_{f}$ remains theoretical; however, its inclusion highlights the flexibility of the framework and prepares the IME for future multicenter validation studies stratified by demographic variables.

The weighting of the domain was guided by evidence that indicates that glabellar frowning and oral commissure depression disproportionately influence the perception of negative affect and social aversiveness [2,12]. Consequently, the relative weights assigned to each domain (glabella, periocular, oral commissure, and frontalis–eyebrow) are based on clinical salience and expert reasoning. These weights should be regarded as provisional priors and will require empirical refinement through future validation studies.

Although the IME is described using formal mathematical expressions, the underlying calculations are intentionally straightforward and suitable for automation within digital tools or clinical software. The mathematical formalization is presented to ensure transparency and reproducibility rather than to imply manual computation in routine clinical practice. For clarity, IME values are expressed as normalized scores (0–1) within formulas and tables, and as a scale 0–100 for clinical interpretation and visualization.

### 2.8. Interpretation Bands

For clinical usability, IME values are categorized into three interpretation bands:

- IME<60: expressive imbalance with negative-valence predominance;
- IME=60–80: harmony zone;
- IME>80: optimized positive valence.

These thresholds are proposed to facilitate clinical communication and longitudinal follow-up, rather than to define diagnostic cut-off points, and should be empirically refined during validation studies.

### 2.9. Personalized Planning (Plan Score)

The IME framework is designed to guide therapeutic prioritization rather than prescribe fixed treatment protocols. Domains with the lowest subscores are identified as candidates for selective neuromodulation, guiding the relative prioritization of the intervention rather than mandating specific therapeutic actions.

Specific doses, injection points, or product-dependent parameters are not defined within this conceptual model. All treatment decisions must adhere to the labeling of the product and the published international consensus guidelines on the use of botulinum toxin [5]. The Plan Score thus serves as a decision-support construct, translating functional assessment into personalized treatment priorities without constraining clinical judgment.

### 2.10. Longitudinal Workflow: The IME Loop

The IME Framework is operationalized through a cyclical workflow designed to support reproducibility and adaptive refinement over time. The IME Loop consists of four sequential phases:

1. Scan: standardized photographic and video acquisition;
2. Score: computation of domain subscores and global IME;
3. Plan: selection and prioritization of domains for intervention;
4. Feedback: reassessment after 2–4 weeks and at regular follow-up intervals.

This iterative loop allows the IME to function as a dynamic marker of expressive equilibrium, supporting longitudinal monitoring and treatment adjustment without prescribing fixed therapeutic protocols. A detailed description of the structural components and the computation of the IME is provided in Table 3, while Table 4 summarizes its interpretative logic and operational use within the proposed framework. A one-page clinical checklist and an example of a feasible consultation workflow are provided in Supplementary Tables S1 and S2.

### 2.11. Hypothetical Case Illustrations

No patient data was collected, and no real clinical cases are presented; the following examples are purely hypothetical and are provided to illustrate how the IME Framework may translate standardized observations into structured scoring and conceptual therapeutic prioritization.

#### 2.11.1. Case 1: Early Hypertonus Without Established Wrinkles

Profile: 32-year-old woman; main complaint: “angry look” despite the absence of major static lines.

- Glabella: FRS =2, FDHS =3⇒Hypertonus=5/8; GLSS =1⇒${\mathrm{IME}}_{G}=0.475$.
- Periocular: Hypertonus=3/8; CFSS =0⇒${\mathrm{IME}}_{P}=0.225$.
- Oral commissure: Hypertonus=1/8; commissural scale =0⇒${\mathrm{IME}}_{C}=0.075$.
- Frontalis–eyebrow: elevation =10/11/9 mm (≈0.83 normalized), FLSS =1 (${\mathrm{FD}}_{\mathrm{Skin}}=0.75$), ESPS =3 (=1.0) ⇒${\mathrm{IME}}_{F}=0.852$.

Global IME:

(9) $$\begin{array}{cc} \mathrm{IME}=100\;\times & (0.30\cdot 0.475+0.20\cdot 0.225+0.40\cdot 0.075+0.10\cdot 0.852)\approx 30.2. \end{array}$$

Interpretation: Marked imbalance dominated by glabellar overactivity despite preserved frontalis function.

Clinical implication (conceptual): Selective neuromodulation of corrugator/procerus only, avoiding unnecessary treatment of elevators or smile-related muscles.

Patient communication (example): “Your facial tension is concentrated between the brows. By selectively relaxing that area, we restore a more balanced and approachable expression”.

#### 2.11.2. Case 2: Periocular Hypertonus with“Tired Eyes”

Profile: 45-year-old man; principal complaint: periocular lines and fatigued appearance.

- Glabella: Hypertonus=3/8; GLSS =2⇒${\mathrm{IME}}_{G}=0.425$.
- Periocular: Hypertonus=6/8; CFSS =3⇒${\mathrm{IME}}_{P}=0.750$.
- Oral commissure: Hypertonus=2/8; commissural scale =1⇒${\mathrm{IME}}_{C}=0.250$.
- Frontalis–eyebrow: elevation =8/9/7 mm (≈0.75 normalized), FLSS =2 (${\mathrm{FD}}_{\mathrm{Skin}}=0.50$), ESPS =2 (≈0.67) ⇒${\mathrm{IME}}_{F}=0.667$.

Global IME:

(10) $$\begin{array}{cc} \mathrm{IME}=100\;\times & (0.30\cdot 0.425+0.20\cdot 0.750+0.40\cdot 0.250+0.10\cdot 0.667)\approx 44.4. \end{array}$$

Interpretation: Moderate imbalance dominated by periocular hypertonus.

Clinical implication (conceptual): Targeted treatment of orbicularis oculi while preserving zygomaticus and frontalis to maintain natural smile dynamics and gaze elevation.

Patient communication (example): “Overactivity around your eyes produces a tired look. By rebalancing that muscle group, we can refresh your gaze without compromising your natural expressivity”.

#### 2.11.3. Case 3: Commissural Depression with Brow Ptosis

Profile: 60-year-old woman; principal complaint: “dull look” due to drooping of the oral cavity and mild brow descent.

- Glabella: Hypertonus=4/8; GLSS =2⇒${\mathrm{IME}}_{G}=0.500$.
- Periocular: Hypertonus=2/8; CFSS =1⇒${\mathrm{IME}}_{P}=0.250$.
- Oral commissure: Hypertonus=6/8; commissural scale =3⇒${\mathrm{IME}}_{C}=0.750$.
- Frontalis–eyebrow: elevation =5/6/4 mm (≈0.42 normalized), FLSS =3 (${\mathrm{FD}}_{\mathrm{Skin}}=0.25$), ESPS =1 (≈0.33) ⇒${\mathrm{IME}}_{F}=0.355$.

Global IME:

(11) $$\begin{array}{cc} \mathrm{IME}=100\;\times & (0.30\cdot 0.500+0.20\cdot 0.250+0.40\cdot 0.750+0.10\cdot 0.355)\approx 53.6. \end{array}$$

Interpretation: Significant imbalance driven by commissural hypertonus combined with reduced frontalis–eyebrow function.

Clinical implication (conceptual): Reduce depressor anguli oris activity to relieve commissural depression while preserving frontalis function to avoid further brow descent; consider minor glabellar adjustment if clinically warranted.

Patient communication (example): “The downward pull of the corners of the mouth and the reduced elevation of the brow contribute to a sad appearance. By reducing the downward forces and protecting the elevators, we restore a more open and positive expression”. The hypothetical case scoring is summarized in Table 5 for a comparative overview.

### 2.12. Synthesis of Conceptual Outcomes

**Figure 1:**
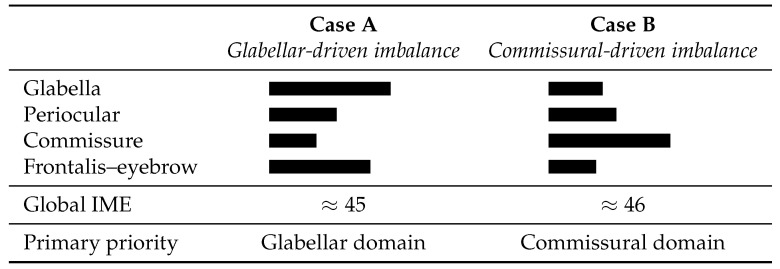
Conceptual illustration showing how different domain-level distributions may yield similar global IME values while leading to distinct interpretive logic and therapeutic prioritization. Although both hypothetical cases present comparable global IME scores, Case A is dominated by glabellar hypertonus with preserved positive-valence function, whereas Case B is driven by commissural imbalance combined with reduced frontalis–eyebrow contribution. The figure illustrates the added value of the IME as an integrative decision-support tool beyond structured qualitative observation alone.

These hypothetical scenarios illustrate how the IME Framework translates qualitative observation into structured scoring and conceptual decision-making. Figure 1 provides a conceptual illustration of how different domain-level distributions can produce similar global IME values while leading to different interpretive logic and therapeutic prioritization. The figure highlights how the composite index integrates heterogeneous inputs beyond structured qualitative observation alone. Although areas of imbalance are often qualitatively apparent on inspection, the IME formalizes their relative contribution across domains, enabling standardized comparison and prioritization even when global severity appears similar.

- Early imbalance (Case 1): detects disproportionate contraction before static wrinkles dominate, supporting minimal and selective intervention.
- Midlife imbalance (Case 2): localizes overactivity to the periocular domain, reducing the likelihood of unnecessary treatment in other regions.
- Advanced imbalance (Case 3): captures the combined contribution of lower-face depressors and reduced upper-face elevator function, supporting a dual correction strategy.

In all cases, the IME provides a reproducible marker of expressive balance. Treatment success is reframed as the maintenance of harmony over time, assessed through numerical indices and standardized visual comparisons, rather than as the pharmacological duration of muscle blockade. In particular, cases with similar global IME values may reflect substantially different internal domain distributions. The global threshold identifies the presence of imbalance, whereas the domain-level subscores determine the location and therapeutic prioritization. This separation between detection and prioritization represents a core functional contribution of IME beyond structured observation alone.

## 3. Discussion

Facial neuromodulation can be reconceived as the management of muscular equilibrium rather than as a mere suppression of the cutaneous lines. This perspective is grounded in converging evidence from psychophysiology and affective neuroscience demonstrating that specific facial muscles are differentially recruited as a function of emotional valence. In particular, the activity of corrugator supercilii reliably increases in association with negative affect, while activation of the zygomaticus major covaries with positive affective states [1,2].

Experimental studies further suggest that altering the ability to frown can modulate central affective processing and subjective mood, consistent with contemporary formulations of the facial feedback hypothesis [12,13,14,15]. Within this evidentiary context, the IME Framework provides a structured and integrative pathway—from anatomical mapping to standardized measurement, decision support, and longitudinal feedback—that links functional facial anatomy, validated photonumeric scales, and newly proposed clinical indices into a coherent approach to aesthetic neuromodulation.

### 3.1. Logical Rationale and Sequencing

The rationale of the IME Framework is described in five sequential steps. First, the Valence Map classifies facial muscles according to their predominant contribution to emotional valence, distinguishing negative-valence depressors from positive-valence elevators. This classification is not an aesthetic preference, but an operational hypothesis: the dominance of depressor activity communicates tension, fatigue, or threat, while the preservation of elevator function supports openness and affiliative intent.

Second, the framework requires measurement of the imbalance as it manifests at rest and during volitional expression. To this end, static and dynamic hypertonus is scored separately using the Face Repose Tension Scale (FRS) and the Facial Dynamics Hypertonus Scale (FDHS), while validated photonumeric scales (GLSS, CFSS, FLSS, and commissural measures) are incorporated to maintain continuity with the regulatory and clinical literature [5,6,7].

Third, these heterogeneous observations are integrated into the Index of Muscular Equilibrium (IME), a standardized score scaled to 0–100 that assigns greater weight to muscular activity (60%) than to line severity (40%). This weighting represents a theoretically motivated a priori decision: it privileges the motor substrate that carries expressive meaning while still acknowledging its cutaneous correlate.

Fourth, the framework explicitly specifies a Frontalis–Eyebrow domain to capture the positive-valence function, including the amplitude of brow elevation, the display of forehead skin, and the symmetry and position of the eyebrows. This design choice avoids the common pitfall of treating elevators in the upper-face solely as structures that should be weakened, despite their communicative role.

Finally, the Plan Score and the IME Loop translate measurement into a reproducible clinical workflow: quantify imbalance, plan selectively, and re-measure using the same standardized protocol at 2–4 weeks and across subsequent cycles.

### 3.2. Relationship to Conventional Paradigms

The IME Framework remains compatible with current aesthetic practice insofar as it relies on validated assessment scales and recognizes established treatment targets such as the glabella, periocular region, and oral commissure. Its primary departure is conceptual rather than procedural.

Traditional paradigms have emphasized wrinkle counts and the duration of muscular blockade as primary markers of success. In contrast, the present model defines success as the restoration and maintenance of expressive harmony, that is, balanced muscular signaling perceived as natural, open, and congruent with the individual’s intended social message. Practically, this reframing replaces the question “How long did the paralysis last?” with “For how long did the patient remain within the harmony zone (IME 60–80) while preserving natural expressivity?”

This distinction is clinically meaningful. Extended paralysis may coincide with reduced naturalness, while sustained harmony may be achieved through smaller selective adjustments anchored to an objective reassessment. By integrating validated severity scales while shifting the interpretive endpoint to function and expressivity, the framework reframes treatment success as time spent within a state of muscular equilibrium rather than the duration of blockade.

The sequencing of the framework reflects this logic. The Valence Map precedes treatment planning because function should guide intervention: depressors associated with negative valence are primary candidates for down-modulation, whereas elevators associated with positive valence should be preserved. Separating static from dynamic hypertonus acknowledges that imbalance may present as baseline tension, exaggerated contraction during expression, or both, each with distinct implications for dosing strategy and risk of over-correction.

The IME then provides a single, interpretable metric that aggregates these inputs while preserving domain-level visibility. Its primary function is not to obscure qualitative assessment, but to formalize how different combinations of hypertonus and cutaneous severity influence prioritization, longitudinal comparison, and treatment logic. The explicit frontalis–eyebrow domain is justified on functional and communicative grounds: brow elevation and symmetric opening are positive-valence signals [2], and their loss—whether iatrogenic or age-related—can degrade perceived vitality. By measuring elevation amplitude, brow position and symmetry, and forehead display, the framework guards against inadvertent over-weakening of elevators, a recognized contributor to “flat” or fatigued appearance.

The feedback loop completes the logic: a metric without longitudinal reassessment is diagnostically incomplete, and treatment without re-measurement is methodologically fragile.

### 3.3. Conceptual Scope of the IME Framework and Role of Extracore Modulators

The IME Framework is specifically designed to quantify facial expressive equilibrium by focusing on mimetic muscle groups that directly contribute to emotional signaling and communicative valence. Consequently, its core computational domains are restricted to high-resolution facial units whose activity modulates perceived affect during both repose and voluntary expression, including glabellar, periocular, commissural, and eyebrow dynamics.

Within this conceptual structure, the platysma occupies a distinct functional role. Although clinically relevant in aesthetic medicine and routinely aimed at improving neck contour and jawline definition, platysmal activity exerts a primarily caudal and contextual influence on facial appearance rather than directly encoding discrete emotional expressions. Its neuromodulation modifies the biomechanical environment of the lower face by reducing downward traction and tension, but does not independently alter expressive valence.

The clinical relevance of platysmal activity in cervico-facial aesthetics is well established, with validated photonumeric and clinical grading systems available for both band severity and neck contour evaluation [16,17]. However, despite their value in treatment planning and outcome documentation, these instruments target contextual contour and tension rather than expressive valence and are therefore intentionally excluded from the computational core of the IME Framework.

For this reason, the platysma is not included as a primary computational variable within the IME score, nor is it introduced as a mathematical modulator of the index. Incorporating platysmal activity into the IME equation would risk conflating contextual contour enhancement with expressive balance, thereby compromising the semantic integrity of the index as a measure of facial equilibrium rather than generalized aesthetic improvement.

Instead, platysmal assessment is conceptualized as an extracore modulatory factor that informs clinical interpretation and personalized treatment planning without directly influencing the IME computation. This distinction preserves the theoretical coherence of the framework while allowing for future modular extensions, such as dedicated cervico-facial equilibrium indices, to explore the interaction between expressive dynamics and caudal tension in a methodologically independent manner.

This modular separation is intentional and preserves the semantic integrity of the IME as an index of expressive equilibrium rather than a generalized aesthetic severity score. Extracore muscles may be incorporated in future extensions of the framework as independent modules, without altering the computational structure of the core IME domains.

### 3.4. Interpretability and Clinical Communication

A recurring limitation in aesthetic practice is the gap between clinical reasoning and patient understanding. The IME Framework addresses this gap by pairing a quantitative index with visual outputs, including the Valence Map and a color-coded IME gage. Together, these tools translate a complex, multi-domain evaluation into a compact narrative that can be revisited over time, facilitating informed consent, expectation alignment, and documentation.

Although designed to support communication, these outputs are not merely didactic. Because they arise from standardized acquisition and predefined scales, they also serve as anchors for quality assurance and inter-rater comparison.

### 3.5. Methodological Considerations and Psychometrics

Three assessment instruments introduced in this framework, FRS, FDHS, and ESPS, are conceptual and require formal psychometric validation. A stepwise validation roadmap is described in Table 6. Key properties to establish include inter- and intra-rater reliability, test–retest stability under standardized acquisition, and responsiveness to clinically meaningful change.

Establishing a minimal clinically important difference (MCID) for the IME and for domain subscores would further aid interpretation in both clinical practice and research. Convergent validity should be assessed against established patient-reported outcome measures, such as FACE-Q Aesthetics [11], and, where feasible, against objective correlates including surface electromyography or kinematic measures during standardized tasks.

The proposed weighting scheme (60/40 for hypertonus versus line severity; 0.30/0.20/0.40/0.10 across domains) is provisional, grounded in clinical importance and theoretical reasoning, and should be empirically refined once sufficiently large datasets become available.

### 3.6. Duration of Harmony Versus Duration of Blockade

A central conceptual contribution of the IME Framework is the relocation of the clinical endpoint from pharmacological paralysis to expressive equilibrium, a distinction that underlies the assessment logic of the framework, the longitudinal monitoring strategy and the ethical positioning. The duration of blockade is treated as an input to the clinical process rather than as its primary goal.

By measuring IME at baseline, early follow-up, and maintenance visits, clinicians can quantify the time spent within the harmony zone, a parameter that may better capture the patient-perceived success of neuromodulation. This approach supports nuanced maintenance strategies, such as smaller, more frequent adjustments versus larger, less frequent ones, tailored to individual dynamics rather than fixed calendars.

Operationally, “duration of harmony” can be defined as the time interval during which a patient remains within the IME harmony zone (60–80). In future longitudinal studies, this parameter could be analyzed using time-to-event methods, defining failure as the first occurrence of IME <60.

### 3.7. Future Refinements

Population variability represents an important avenue for future refinement. Facial expressivity and the perception of emotional valence vary between cultures, age groups, and sex [1]. Although the present framework introduces a theoretical population calibration factor ($C_{f}$), prospective multicenter studies will be required to derive validated coefficients. Such calibration would allow the IME to be adapted across diverse populations while maintaining reproducibility and objectivity.

### 3.8. Limitations

This work is intentionally limited to a conceptual and methodological contribution. Consequently, the IME Framework does not specify treatment doses, injection points, or product-dependent parameters. Such variables are known to strongly influence clinical outcomes, but are intentionally positioned outside of the scope of the IME to preserve its generalizability and conceptual focus on assessment, prioritization, and longitudinal monitoring. It does not propose or validate clinical decision thresholds, dosing algorithms, or treatment protocols, and no patient data was collected. The IME thresholds and weights, although theoretically rational, await empirical confirmation and may require refinement through prospective multicenter studies.

Clinical implementation also entails practical constraints. Standardized photo and video acquisition adds time to consultations, and inter-rater variability may occur without training. The perception of facial valence is influenced by demographic factors, suggesting that IME cutoffs may require normative calibration. Privacy and data protection must be ensured for the storage and comparison of patient images and videos.

Finally, while the framework is designed to be product-agnostic, clinical use must adhere strictly to the product label and consensus safety guidelines [5,18], and the IME should never replace detailed anatomical expertise [19]. Together, these considerations underscore that the present work represents a testable conceptual model rather than a claim of clinical efficacy.

### 3.9. Future Directions

Several lines of investigation may operationalize and extend the IME Framework. In particular, the model is well suited to interdisciplinary contexts in which aesthetic and functional facial concerns overlap, including facial asymmetry, muscle hyperactivity related to bruxism, and other conditions commonly addressed with botulinum toxin.

Future work may include the following directions:

1. Prospective validation studies to quantify reliability, responsiveness and minimal clinically important difference (MCID) for the IME and its domain subscores. Multicenter designs would improve generalizability.
2. Correlation with patient-reported results, particularly FACE-Q Aesthetics and procedure-specific modules, to establish external validity and patient-centered interpretability of IME changes [11].
3. Digital augmentation through computer-vision pipelines that enable automated facial landmark analysis and displacement analysis (e.g., brow head/body/tail; commissure trajectory), standardized task detection in short videos, and automated domain scores computation. AI-assisted quality control could detect suboptimal lighting, pose, or acquisition artifacts.
4. Objective physiological integration, including surface electromyography [20] to derive intra-subject *z*-scores and corrugator/zygomaticus activity ratios during standardized tasks, as well as ultrasound guidance [21] to support anatomically precise planning in regions with high inter-individual variability.
5. Longitudinal analytics, modeling time spent within the IME harmony zone as a survival-type endpoint, enabling comparison of maintenance strategies beyond simple time-to-waning metrics.
6. Education and standard setting, through the development of annotated atlases and example video libraries to train raters on FRS, FDHS, and ESPS scoring, and the publication of open reference datasets to facilitate external replication.
7. Broader applicability, exploring whether equilibrium-based reasoning may inform adjacent interventions, such as filler strategies that preserve elevator vectors or peri-oral balance, and whether analogous concepts may be informative in rehabilitative settings where facial expressivity is clinically relevant.
8. Conceptual convergence with functional disciplines. Although the IME Framework is intended as an aesthetic-specific tool, its underlying rationale—rebalancing hyperactive and hypoactive muscle groups—parallels approaches used in facial rehabilitation and neuromuscular therapy. Although the present methodology is not directly transferable, this theoretical convergence may foster interdisciplinary dialog and future adaptation.

### 3.10. Ethical and Communicative Implications

By prioritizing muscular equilibrium and transparency of measurement, the IME Framework addresses two recurrent risks in aesthetic neuromodulation: over-treatment driven by the pursuit of prolonged paralysis, and misaligned expectations arising from the conflation of cutaneous smoothness with attractiveness.

A shared clinical language—rooted in function, quantified by the IME, and visualized through standardized outputs—may improve shared decision-making, improve expectation alignment, and reduce dissatisfaction associated with unnatural appearance. In this sense, the framework supports not only technical standardization but also ethical practice, emphasizing proportionality, reversibility, and longitudinal accountability.

In summary, the IME Framework articulates a logically ordered and measurable approach to facial neuromodulation that is consistent with contemporary affective science. It remains compatible with validated clinical scales while introducing testable constructs that foreground muscular balance and expressive authenticity. Although the proposal awaits empirical validation, it offers a unified structure for assessment, planning, and longitudinal evaluation that reframes success as time spent in expressive harmony rather than months of pharmacological paralysis.

## 4. Conclusions

Conceptually, the IME Framework may be summarized as a four-step sequence: (i) functional mapping of facial valence, (ii) standardized measurement of static and dynamic hypertonus, (iii) integration into a composite equilibrium index, and (iv) longitudinal feedback-driven refinement.

It introduces a conceptually grounded approach to aesthetic neuromodulation that shifts the clinical focus from wrinkle suppression and duration of paralysis to the restoration of muscular equilibrium and expressive harmony. By integrating validated wrinkle severity scales with newly proposed indices of hypertonus and positive-valence preservation, the framework provides an objective and reproducible method to quantify facial balance.

The introduction of the Index of Muscular Equilibrium (IME), the Valence Map, and the iterative IME Loop offers a structured pathway for standardized assessment, personalized planning, and longitudinal monitoring. Although the framework is currently conceptual, its rationale is anchored in established neuroscientific and psychophysiological evidence linking facial muscle activity to emotional valence and social signaling.

Future studies will be required to validate the proposed scales, establish the reliability and sensitivity of the IME, and explore correlations with patient-reported outcomes. At its current stage, the IME Framework should be regarded strictly as a conceptual and methodological proposal. It is not intended for routine clinical implementation or commercial application prior to empirical validation, psychometric testing, and prospective outcome studies. If empirically supported, this paradigm may contribute to a rethink of aesthetic neuromodulation, positioning botulinum toxin treatment as a strategy to preserve expressive authenticity and functional harmony rather than to eliminate cutaneous lines solely.

Within this perspective, the IME Framework may also serve as a shared methodological language for future investigations that include aesthetic outcomes and functional facial conditions treated with botulinum toxin.

## Figures and Tables

**Table 1:** Scales used in the IME Framework.

Domain	Scale	Range	Description
Hypertonus (static)	Face Repose Tension Scale (FRS, proposed)	0–4	Muscle tension at rest, ranging from none (0) to marked distortion (4).
Hypertonus (dynamic)	Facial Dynamics Hypertonus Scale (FDHS, proposed)	0–4	Excessive muscular contraction or distortion during standardized expression tasks.
Wrinkles—Glabella	Glabellar Line Severity Scale (GLSS)	0–4	FDA-validated photonumeric photographic scale for glabellar line severity.
Wrinkles—Periocular	Crow’s Feet Severity Scale (CFSS)	0–4	FDA-validated photonumeric photographic scale for periocular wrinkles.
Wrinkles—Forehead	Forehead Line Severity Scale (FLSS)	0–4	Validated scale for frontal line severity assessment.
Oral commissure	Marquardt oral commissure scale/angular measurement	0–4 or °	Severity of commissural depression assessed using geometric facial analysis.
Eyebrow position	Eyebrow Symmetry and Position Scale (ESPS, proposed)	0–3	Symmetry, position, and mobility of eyebrow head, body, and tail.

Legend: Table 1 summarizes the validated and proposed assessment scales integrated into the IME Framework. Proposed instruments (FRS, FDHS, ESPS) are conceptual tools and require psychometric validation in future clinical studies. Assessment of platysmal activity is performed using validated photonumeric scales, but is not included as a primary computational domain within the current IME formulation.

**Table 2:** IME weighting scheme for facial domains.

Domain	Components Included	Weight in Global IME
Glabella	Corrugator and procerus hypertonus combined with glabellar line severity (GLSS).	0.30
Periocular	Orbicularis oculi hypertonus combined with crow’s feet severity (CFSS).	0.20
Oral commissure	Depressor anguli oris hypertonus combined with commissural angular displacement.	0.40
Frontalis–eyebrow	Upper-face equilibrium combining frontalis mobility, forehead skin display, and eyebrow symmetry and position (ESPS).	0.10
Global IME	Weighted sum of all facial domains.	1.00

Legend: Table 2 details the relative weights assigned to each domain in the IME Framework. Weights are provisional and represent theoretically informed initial priors derived from clinical salience and literature on emotional valence in facial expression. Cervico-facial muscles such as the platysma are intentionally excluded from the computational weighting scheme, as they modulate facial context rather than expressive valence.

**Table 3:** IME Loop: detailed longitudinal workflow for standardized monitoring and adjustment.

Phase	Objective	Operational Steps and Outputs
Scan	Acquire standardized static views (frontal, 3/4 right, 3/4 left) and dynamic tasks (neutral repose, frown, closed-lip smile, broad smile with teeth, eyebrow elevation) under controlled lighting, neutral head position aligned to the Frankfort plane, and constant camera distance.	
Score	Quantify hypertonus and cutaneous manifestations; compute domain subscores and global IME	Rate static hypertonus using FRS (0–4) and dynamic hypertonus using FDHS (0–4) per domain. Integrate validated photonumeric scales (GLSS, CFSS, FLSS) and commissural angular measures. Compute negative-valence domain ${\mathrm{IME}}_{d}$ and the positive-valence frontalis–eyebrow domain ${\mathrm{IME}}_{\mathrm{Frontalis}}$, then compute global IME (0–100). Output: domain subscores, global IME, and interpretation band.
Plan	Translate scoring into therapeutic prioritization (decision support)	Identify lowest-performing domains (relative deficits) and define treatment priorities emphasizing reduction of negative-valence depressor activity while preserving positive-valence elevator function. Output: prioritized targets and monitoring plan. Note: No fixed doses or injection points are specified; clinical protocols must follow labeling and consensus guidance.
Feedback	Reassess outcomes and adapt plan iteratively	Reassess at 2–4 weeks post-intervention and at regular intervals thereafter. Repeat Scan and Score; compare changes in domain subscores and global IME; update Plan based on response stability, symmetry, and adverse events (if any). Output: longitudinal trajectory of IME and domain-specific response profiles. Adverse events, if present, are documented separately and are not incorporated into the IME score.

Legend: Table 3 provides an operational specification of the IME Loop intended to support reproducibility and longitudinal adjustment. Outputs are designed to be software-implementable for automated reporting and standardized follow-up.

**Table 4:** IME Loop: compact overview for clinical use.

Step	What Is Done	Typical Timing
Scan	Standardized photo/video acquisition (static + dynamic tasks).	Baseline; each follow-up
Score	FRS/FDHS + validated wrinkle scales (GLSS, CFSS, FLSS) + commissural angular measures; compute domain subscores and global IME.	Immediately after Scan
Plan	Prioritize domains with lowest subscores; define selective neuromodulation strategy (conceptual decision support, non-prescriptive).	
Feedback	Repeat Scan/Score; compare trajectories; refine plan.	2–4 weeks post; then periodic

Legend: Table 4 summarizes the IME Loop as a quick-reference implementation guide. Timing should be adapted to clinical context, indication, and follow-up routines.

**Table 5:** Hypothetical patient cases scored with the IME Framework.

Case	Age/Profile	Domain Scores (0–1)	Global IME	Interpretation
Case 1	32 y, early hypertonus without established wrinkles	G 0.475; P 0.225; C 0.075; F 0.852	0.30	Marked imbalance
Case 2	45 y, periocular hypertonus with “tired eyes”	G 0.425; P 0.750; C 0.250; F 0.667	0.44	Moderate imbalance
Case 3	60 y, commissural depression with mild brow ptosis	G 0.500; P 0.250; C 0.750; F 0.355	0.54	Significant imbalance

Legend: Table 5 summarizes the three hypothetical cases described in the text. Domain scores are normalized (0–1) for the glabella (G), periocular (P), commissure (C), and frontalis–eyebrow (F) domains and combined using the weighting scheme reported in Table 2 to compute a global IME. For consistency with the mathematical formulation of the framework, the global IME is reported here in normalized form (0–1); multiplication by 100 yields the 0–100 scale used for clinical interpretation elsewhere in the manuscript.

**Table 6:** Conceptual roadmap for validation of the IME Framework.

Step	Aim	Design	Key Metrics
1	Reliability of FRS, FDHS, and ESPS	Inter- and intra-rater pilot study under standardized acquisition conditions.	Intraclass correlation coefficient (ICC); Cohen’s kappa.
2	Responsiveness of IME and domain subscores	Prospective pre–post intervention study using botulinum toxin.	Effect size; minimal clinically important difference (MCID).
3	Convergent validity	Correlation of IME and subscores with FACE-Q Aesthetics and other patient-reported outcome measures.	Pearson or Spearman correlation coefficients.
4	Weight refinement	Multivariate modeling in a large, prospectively collected cohort.	Regression-based domain weighting; model fit indices.

Legend: Table 6 outlines a stepwise roadmap for empirical validation of the IME Framework, progressing from reliability testing of newly proposed scales to outcome correlations and refinement of domain weighting.

## Data Availability

No new data were created or analyzed in this study. Data sharing is not applicable to this article.

## Supplementary Materials

The following supporting information can be downloaded at https://www.mdpi.com/article/10.3390/toxins18020115/s1: Table S1: IME Framework—a one-page clinical checklist summarizing the standardized assessment workflow, including domain mapping, scoring tools, IME calculation, therapeutic planning, and longitudinal follow-up; Table S2: Example of a clinical application workflow of the IME Framework, illustrating stepwise actions, required tools, estimated timing, and outputs during a standard consultation.
