# Supplementary material for: From Muscular Hypertonus to Equilibrium: A Conceptual Framework for Aesthetic Neuromodulation Based on the Index of Muscular Equilibrium (IME)
Source: Toxins (Basel). 2026 Feb 23;18(2):115. doi: 10.3390/toxins18020115 (PMC12945272; doi:10.3390/toxins18020115)
Supplement: Supplementary file 1 [file toxins-18-00115-s001.zip › toxins-4072610-supplementary/Supplementary Table S1.pdf]

Supplementary Table S1. IME Framework – One-Page Clinical Checklist

Step	Task	Tool / Scale	Score / Output
**SCAN**	Standardized photo & video (rest + frown + smile + brow raise)	Camera, fixed lighting	Visual dataset
**MAP**	Identify domains (glabella, periocular, commissure, frontalis–eyebrow)	Valence Map	Domains labeled
**SCORE**	Static hypertonus	FRS (0–4)	___ / 4
	Dynamic hypertonus	FDHS (0–4)	___ / 4
	Wrinkles	GLSS, CFSS, FLSS (0–4 each)	___ / 4
	Eyebrow position	ESPS (0–3)	___ / 3
	Calculate IME per domain	Weighted formula	___ / 1
	Global IME score	Weighted sum	___ / 1
**PLAN**	Interpret IME zone	<0.60 = imbalance 0.60–0.80 = harmony >0.80 = hypercorrection	Zone = ____
	Therapeutic plan	Domain-targeted neuromodulation plan (conceptual)	Plan documented
**FEEDBACK**	Post-treatment documentation	Repeat photo/video	Comparative dataset
	Recalculate IME	Same workflow	Follow-up IME = ____
	Monitor longitudinally	Time in harmony (IME ≥0.60)	Duration recorded

Legend: This one-page checklist summarizes the operational workflow of the IME Framework. It allows structured data capture, reproducibility, and easy integration into clinical practice or research settings.
