# Supplementary material for: From Muscular Hypertonus to Equilibrium: A Conceptual Framework for Aesthetic Neuromodulation Based on the Index of Muscular Equilibrium (IME)
Source: Toxins (Basel). 2026 Feb 23;18(2):115. doi: 10.3390/toxins18020115 (PMC12945272; doi:10.3390/toxins18020115)
Supplement: Supplementary file 1 [file toxins-18-00115-s001.zip › toxins-4072610-supplementary/Supplementary Table S2.pdf]

Supplementary Table S2.

Example of Clinical Application Workflow for the IME Framework

Step	Action	Tools/Measures	Estimated Time	Output
1	Standardized photo/video capture (frontal, ¾ views; repose + 3 tasks: frown, gentle smile, full smile)	Controlled lighting, neutral head position	~2 min	Baseline documentation
2	Checklist scoring: hypertonus (FRS + FDHS), wrinkle severity (GLSS, CFSS, FLSS), eyebrow position (ESPS)	Clinical scales, standardized forms	~3 min	Domain-level raw scores
3	Calculation of IME (per domain + global index)	Formula with weighting (hypertonus 60%, line severity 40%, eyebrow position included)	~2 min	Numeric IME (0–100; derived from normalized score) + tachometer visualization
4	Treatment planning	Domain prioritization, dose mapping, adherence to consensus guidelines; extracore modulators (e.g., platysma) considered as clinically indicated	~5–7 min	Personalized neuromodulation plan
5	Patient communication	Visual report: before/after comparables, Valence Map, tachometer-style IME bar	~1–2 min	Patient-facing summary, consent reinforcement

Total estimated time: ~15 minutes per consultation.

Legend

FRS = Face Repose Tension Scale; FDHS = Facial Dynamics Hypertonus Scale; GLSS = Glabellar Line Severity Scale; CFSS = Crow's Feet Severity Scale; FLSS = Forehead Line Severity Scale; ESPS = Eyebrow Shape and Position Scale; IME = Index of Muscular Equilibrium.

This table illustrates a typical clinical workflow of the IME Framework during a standard consultation. The process is divided into five operational steps, each with approximate timing,

required tools, and outputs. The goal is to demonstrate feasibility within routine aesthetic practice (~15 minutes), while ensuring standardization, reproducibility, and patient communication.
